# Innovative approaches to integrating gender into conventional maize breeding: lessons from the Seed Production Technology for Africa project

**DOI:** 10.3389/fsoc.2023.1254595

**Published:** 2023-09-19

**Authors:** Rachel C. Voss, Jill E. Cairns, Michael Olsen, Francisca Ndinda Muteti, George Magambo Kanyenji, Esnath Hamadziripi, Dickson Ligeyo, Kingstone Mashingaidze, Sarah Collinson, Susan Wanderi, Vincent Woyengo

**Affiliations:** ^1^Sustainable Agri-food Systems Program, International Maize & Wheat Improvement Center (CIMMYT), Nairobi, Kenya; ^2^Global Maize Program, International Maize & Wheat Improvement Center (CIMMYT), Harare, Zimbabwe; ^3^Global Maize Program, International Maize & Wheat Improvement Center (CIMMYT), Nairobi, Kenya; ^4^Kenya Agricultural and Livestock Research Organization (KALRO), Nairobi, Kenya; ^5^Agricultural Research Council (ARC), Pretoria, South Africa; ^6^Corteva Agriscience, Woodland, CA, United States

**Keywords:** gender, crop breeding, on-farm trials, social inclusion, tricot, citizen science

## Abstract

The integration of gender concerns in crop breeding programs aims to improve the suitability and appeal of new varieties to both women and men, in response to concerns about unequal adoption of improved seed. However, few conventional breeding programs have sought to center social inclusion concerns. This community case study documents efforts to integrate gender into the maize-focused Seed Production Technology for Africa (SPTA) project using innovation history analysis drawing on project documents and the authors’ experiences. These efforts included deliberate exploration of potential gendered impacts of project technologies and innovations in the project’s approach to variety evaluation, culminating in the use of decentralized on-farm trials using the tricot approach. Through this case study, we illustrate the power of active and respectful collaborations between breeders and social scientists, spurred by donor mandates to address gender and social inclusion. Gender integration in this case was further facilitated by open-minded project leaders and allocation of funding for gender research. SPTA proved to be fertile ground for experimentation and interdisciplinary collaboration around gender and maize breeding, and has provided proof of concept for larger breeding projects seeking to integrate gender considerations.

## 1. Introduction

Gender integration in breeding programs responds to concerns that men and women are not taking up new crop varieties at equal rates (Orr et al., 2018; Tufan et al., 2018; CGIAR Excellence in Breeding Initiative, 2020). Crop breeding programs may seek to address gender gaps in variety uptake either through gender-responsive breeding or gender-intentional breeding. While the former involves assessing gender-based differences in farmers’ needs, priorities, and constraints, and monitoring and mitigating any negative gendered impacts, the latter may involve deliberately developing varieties that directly benefit women and/or address gender inequalities (Ashby and Polar, 2021).

The gender gap in uptake of improved varieties appears to hold true for maize seed in Africa, as illustrated in comparisons of male- and female-managed plots or male- and female-headed households (Kassa, 2013; Fisher and Kandiwa, 2014; Fisher and Carr, 2015; Fisher et al., 2019). This raises questions about whether maize breeding programs in Africa, which involve national agricultural research and extension organizations (NARES), the private sector, and CGIAR research centers, are adequately responding to the needs and priorities of women farmers (Voss et al., 2021). Much of the vanguard work on gender and breeding has focused on beans, roots, and tubers (Orr et al., 2018; Tufan et al., 2018), while maize breeding programs have arguably lagged behind. This may be linked to the CGIAR Gender and Breeding Initiative’s initial priorities; women’s outsized role in the production of tuber and legume crops relative to maize, which is more often jointly managed (Voss et al., 2023); and evidence that gender-based differences in maize preferences are less clear than for other crops (Voss et al., 2021).

Despite the high interest in gender integration into major conventional breeding programs such as maize (CGIAR Excellence in Breeding Initiative, 2020; Bill and Melinda Gates Foundation, 2021), this process presents challenges. The pursuit of either gender-responsive or gender-intentional breeding often requires a reordering of priorities and reallocation of resources within breeding institutions, which could have implications for breeding efficiency. It is therefore of critical interest to understand how these reforms can be realized in large, well-established breeding programs.

In the case study that follows, we document the process of gender integration into a centralized maize breeding project in eastern and southern Africa (ESA). For this, we use an innovation history analysis approach (Douthwaite and Ashby, 2005). We draw on the authors’ personal experiences and project materials to document an innovative recent project, reflect on success and challenges, and identify lessons learned. We ground this case study in institutional and structuration theories as we examine processes of institutional change as a function of actions and interactions between actors within breeding programs (Barley and Tolbert, 1997). Through this analysis, we document how negotiations between actors within breeding programs can shift scripts, expectations, and behaviors in a way that create space for institutional innovation around gender and breeding.

## 2. Context

Women’s heavy involvement in maize production in Africa and its general importance as a food source underscore the relevance of gender integration in maize breeding. Maize is widely grown as a staple crop in ESA, typically by both men and women, and often for both household consumption and commercial sales. Crop management varies by locale, with men and women independently cultivating maize in some regions with larger farm sizes, e.g., Zimbabwe (Cairns et al., 2021a), and jointly cultivating maize with their spouses in other regions, e.g., Kenya (Voss et al., 2023). In Tanzania and Mozambique, the family as a whole is often involved in land preparation, weeding, harvesting, and threshing of maize, although specific tasks can skew toward women or men depending on the context (Adam et al., 2020a,b).

Studies of maize systems in ESA highlight differences in management and gender gaps in productivity between male- and female-managed plots and male- and female-headed households. In Zimbabwe, significant differences were found in variety choice, use of intercropping, and recycled seed use between men’s and women’s maize plots (Cairns et al., 2021a). Women’s plots in maize-growing regions have also been shown to be less productive than men’s (Cairns et al., 2021b). Productivity gaps are generally attributed to men’s advantages in accessing and controlling resources, including fertile land (Burke et al., 2018; Burke and Jayne, 2021), fertilizer (Adam et al., 2021), labor and labor-saving technologies (Andersson Djurfeldt et al., 2019), and information about new technologies (Fisher et al., 2019).

Efforts to mainstream gender in maize breeding programs in Africa began in earnest in 2015, when a large project at the International Maize and Wheat Improvement Center (CIMMYT) set out to incorporate gender-preferred traits into the maize breeding pipeline. The breeding team, with minimal training around gender and limited guidance from social scientists and gender researchers (for whom turnover in this period was high), started to routinely solicit gender-disaggregated preferences in on-farm trials to identify traits relevant for women. Collection of preference and adoption data became standard, frequently through gender-disaggregated studies of variety adoption (Fisher et al., 2015, 2019) or participatory varietal selection (PVS). In PVS, breeders and social science collaborators invited women and men farmers to regional varietal trials, which were “on-farm,” but for which researchers supplied inputs. Farmers invited to visit these trials typically scored up to 20 varieties on a range of traits, and breeding teams used these data to validate advancement decisions and assess preferences along gender lines (Setimela et al., 2017; Worku et al., 2020). Because trial-hosting farmers often wanted to demonstrate their skills and field agents’ priority was to execute successful trials with reliable farmers, this type of on-farm trial was not optimal for allowing a diversity of farmers to individually test and evaluate varieties in their farm environments. Fundamentally, neither PVS nor adoption studies have generated clear or consistent insight into gender-based preferences to guide gender-responsive or -intentional maize breeding (Voss et al., 2021).

## 3. Details: Seed Production Technology for Africa project

It was in this context that the Seed Production Technology for Africa project (SPTA) launched at CIMMYT in 2016, in follow up to the Improved Maize for African Soils (IMAS) project, with a second phase (SPTA2) funded in 2020. In collaboration with Corteva AgriScience, SPTA evaluated the use of a non-pollen producing maize gene, *Ms44*, to reduce the complexity of hybrid maize seed production by removing the need to detassel female parents in seed production. Hybrids produced using *Ms44* segregate 1:1 for pollen producing and non-pollen producing plants and are thus referred to as 50% non-pollen producing (FNP). This technology offers three key benefits: (1) increased female seed yield because detasseling is unnecessary, (2) improved quality assurance during seed production, and (3) increased yield of FNP hybrids due to reduced tassel growth and greater partitioning of nitrogen to the ear, increasing nitrogen use efficiency (NUE; Fox et al., 2017).

The SPTA team at CIMMYT was smaller than many breeding projects. The project leader (PL), a male breeder, was highly committed to serving the most vulnerable farmers. In previous work in the private sector, he had engaged superficially with market segmentation research, and was greatly inspired by a CGIAR Gender Platform workshop on farmer typologies and gender (where he reported being one of the few breeders in the room). This engagement motivated him to consider not only standard genotype-by-environment interactions, but genotype-by-environment-by-farmer interactions. He had also been deeply influenced by a colleague’s presentation on the gender gap in use of improved maize varieties. Although he had long been familiar with the “gender narrative,” the robust and compelling econometric data she shared drove home the relevance of gender. During SPTA’s implementation, the PL made it a priority to visit a subset of on-farm trials every year and speak to household members, which further underscored concerns around gender, labor, and resource access.

The primary breeding team also involved two female scientists (one of whom was deeply interested in gender issues) and three male breeders (from CIMMYT and NARES). Two female gender researchers at CIMMYT worked on the project consecutively alongside several male social scientists. These team members regularly exchanged knowledge and ideas informally in monthly project meetings and together worked to center gender concerns.

### 3.1. Early recognition of potential gendered impacts

The PL, having had some exposure to gender and breeding work, quickly recognized the potential implications of the *Ms44* gene for women and other resource-constrained farmers in Africa. Early research in the United States showed FNP hybrids had a higher yield than their pollen-producing pairs under sub-optimal nitrogen levels, suggesting relevance for low-fertility soils and low-input systems (Fox et al., 2017). The project proposals for SPTA’s precursor, IMAS, discussed the genetic technology’s anticipated benefits to women and resource-poor farmers but outlined no plans for collection and analysis of gender data, nor did it involve any social scientists.

In 2013, donors to the CGIAR MAIZE Research Program mandated a “gender audit” of the wider breeding program. The resulting report emphasized that while many CIMMYT projects engaged superficially with gender concerns, gender data and analysis were often lacking. The SPTA PL thus advocated for funding for gender research in SPTA, leveraging the gender audit report and the Bill & Melinda Gates Foundation’s new mandate that the breeding projects they fund address gender. The SPTA proposal included funding for a gender specialist to collect and analyze gender data on preferences and assess potential social and economic impacts.

**Figure 1:**
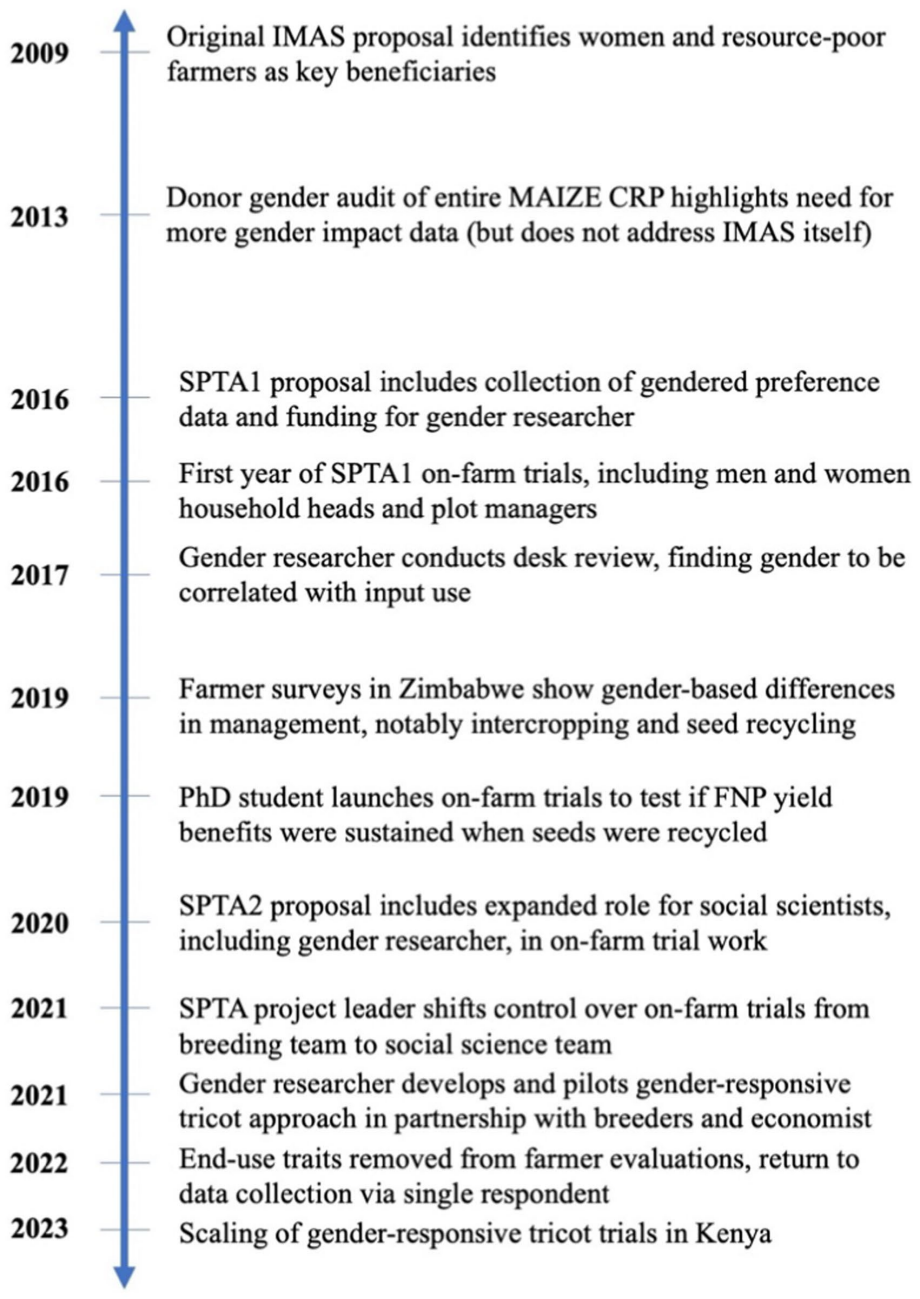
Timeline of gender integration into Seed Production Technology for Africa (SPTA) activities.

At the start of SPTA1, the gender researcher conducted a review underscoring that women in sub-Saharan Africa typically use less fertilizer than men (Adam et al., 2021). This challenge is frequently compounded by women’s cultivation of smaller plots (Udry, 1996; Chirwa et al., 2011; Kilic et al., 2013) with lower quality soils (Ndiritu et al., 2014; Burke et al., 2018; Burke and Jayne, 2021). This review led the breeding team to explicitly target women and resource-poor farmers as end-users; the SPTA technology offered the possibility to increase yields on women’s fields without the requirement that they increase fertilizer use—which, while desirable, is not always feasible—and increase the return on fertilizer investments. Understanding any gender-based differences in the performance of or preferences for new FNP maize varieties became a priority and led to pronounced shifts in the project’s approach to variety evaluations (Figure 1).

### 3.2. Making variety trials more responsive to farmer realities and gender

A critical first step for SPTA was to validate the yield benefit of the FNP trait in low-input environments in ESA. In the first year of SPTA1, the PL judged that varieties had been adequately validated on-station, so resources for on-station trials were re-allocated to several dozen on-farm trials managed by the breeding team. The first year of data from on-farm testing in Kenya (2016) showed that yield levels in the trials were significantly higher than average for the area. This is a familiar problem in on-farm trials, which have been scrutinized for input and yield levels that are not representative of most smallholders’ realities (De Roo et al., 2017; Laajaj et al., 2020). In this case, discussion between project breeders and extension agents revealed that many farmers were treating trials as demonstration plots and prioritizing them with their own fertilizer inputs.

As such, the SPTA team adjusted the on-farm trial protocols to better enable evaluation under reduced inputs levels. In the second year of expanded on-farm trials (2017), farmers were given seed and fertilizer specifically to create a dedicated demonstration plot, separate from their trial plot where clear protocols ensured yield levels would be more representative. To satisfy basic gender inclusivity requirements, trials were hosted by both male and female household heads and plot managers. However, some tensions emerged around trials with female plot managers in cases where their husbands did not support the handover of control over trials, likely due to social norms around household headship and decision-making. Attentive members of the breeding team recognized that they had created unintended pressures on the women involved by overlooking household power dynamics. The project team saw this as an important learning opportunity, highlighting the need for more sensitivity to gender dynamics in on-farm trial execution.

These early on-farm assessments of FNP hybrids in large-scale trials showed a yield benefit of 200 kg ha^−1^ across yield levels, translating to a larger proportionate yield increase for farmers with the lowest yields (Collinson et al., 2022). Farmer evaluations in on-farm trials were conducted using gender-disaggregated PVS. These assessments showed acceptance, across genders, of the FNP trait; although farmers noticed differences in tassel and pollen formation between FNP hybrids and conventional hybrids, they favored FNP hybrids overall due to the improved ear size and increased yield (De Groote et al., 2023).

### 3.3. Revamping the on-farm trial approach

Members of the SPTA team had repeatedly observed that women’s management and variety choices appeared to differ meaningfully from men’s, despite similarities in *stated* preferences. In 2019, SPTA team members in Zimbabwe conducted a study on gender and maize management in partnership with a male systems agronomist. They found significant gender-based differences in management practices, including wider use of intercropping and recycled seed on female-managed plots and in female-headed households (Cairns et al., 2021a). The study also showed discrepancies between farmers’ stated preferences in PVS evaluations and varieties they used at home—especially among women. These findings highlighted, first, that although SPTA’s improved on-farm trial design enabled evaluation of FNP hybrids under realistic input levels, the prescription of other management practices might have unintentionally excluded agronomic practices used disproportionately by women. This led the breeding support specialist to advocate for two actions: to study the yield benefit associated with the FNP trait when seeds were recycled, in partnership with a female Ph.D. student, and to explore new approaches to on-farm trials that could enable variety evaluations under farmers’ preferred management practices.

Second, the survey indicated that PVS-based evaluations of farmer preferences were not adequate to predict real-world demand for varieties. As the core questions of varietal performance in SPTA had been answered, the PL felt that the central challenge for the project was understanding what farmers would actually purchase. This would require a new approach to on-farm trials that would treat them as real-world testing grounds where resource-poor farmers could more directly evaluate new varieties. The PL believed seed demand was best assessed by economists and other social scientists, so the second phase of SPTA included modest funding for a gender researcher and economists to support research on demand creation. The PL also shifted responsibility for on-farm trials to the social science team with inputs from the breeding team.

**Figure 2:**
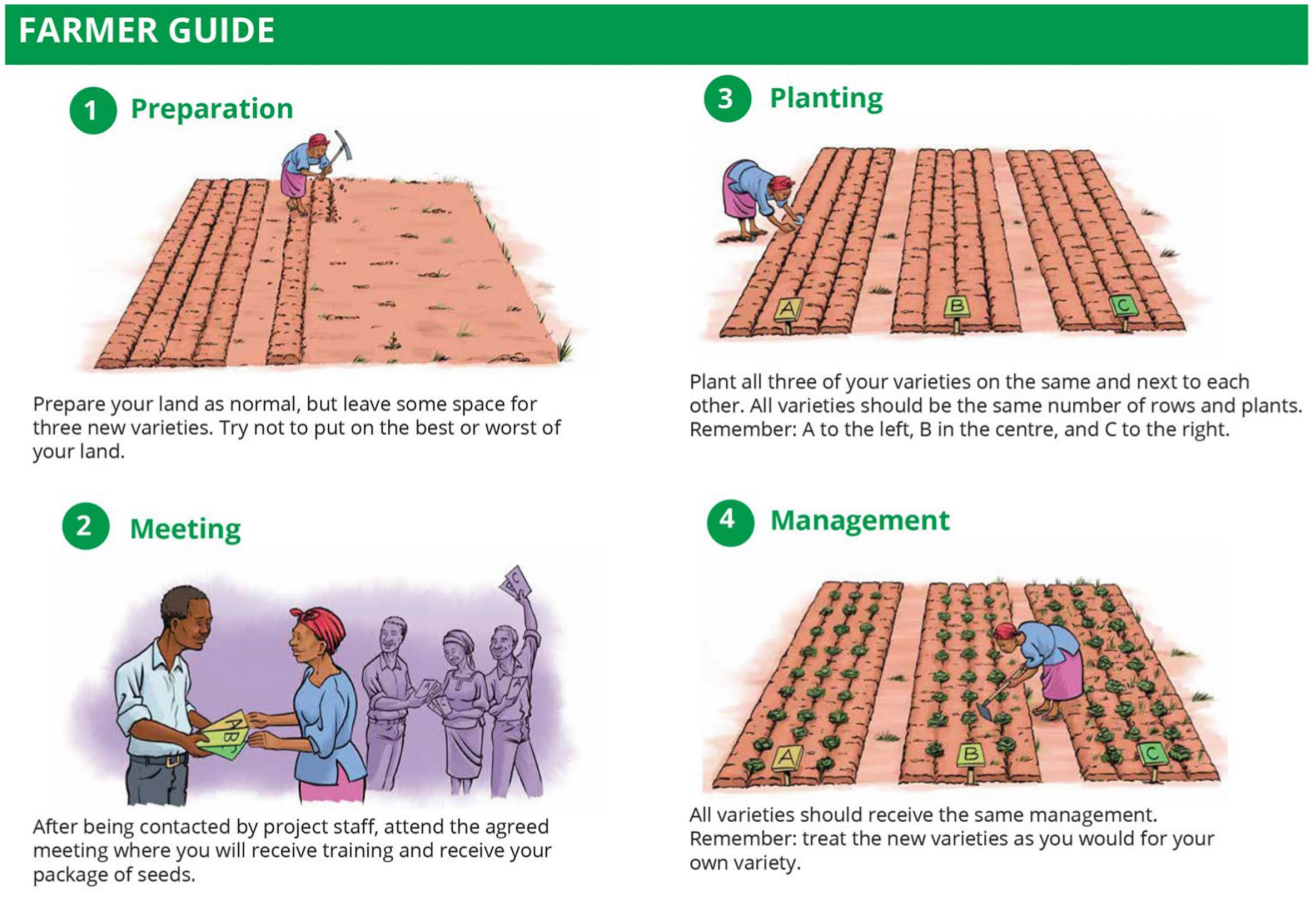
Participants in on-farm tricot trials received visual guidance emphasizing that they should practice their preferred management within the trial setup. Image provided by the 1000FARMS and Scaling Tricot projects: https://climmob.net/blog/wiki/graphic-resources/.

The PL, having read about the triadic comparison of technologies (“tricot”) approach to on-farm trials (van Etten et al., 2019), worked with the gender researcher and other members of the breeding team to develop a revised trial protocol. Building on previous experiences in participatory variety selection, the tricot approach engages a large number of “citizen scientists” to evaluate technologies under representative crop management conditions chosen by the farmer, using incomplete blocks of three varieties, with digital support throughout the process (van Etten et al., 2020). This approach minimizes researcher control, allows farmers with limited land to participate, and enables participants to test technologies on land they know well and make observations throughout the season. Although trial yields are often lower and more variable when researcher involvement is limited (Kool et al., 2020; Laajaj et al., 2020), the tricot approach allows for a large number of trials to be conducted within a set budget, and can thereby compensate for less robust data (Figure 2).

The SPTA team piloted an on-farm trial methodology following the tricot approach in 2021 with 112 farmers (55% women) in Kenya. This approach enabled farmer evaluations that were grounded in men’s and women’s personal realities, including their labor contributions, land quality, and input access. The SPTA team provided participating farmers with a set number of maize kernels (200 per variety) and only basic guidance, i.e., to plant trials in the middle of their maize field, away from trees, and ensure relatively consistent growing conditions (e.g., slope and soil type) among subplots. Otherwise, farmers were requested to practice their preferred management, including intercropping, but to apply consistent crop management across subplots. The team collected spacing data from farmers and field agents to estimate yields and genetic gains in lieu of standardizing planting arrangements.

**Figure 3:**
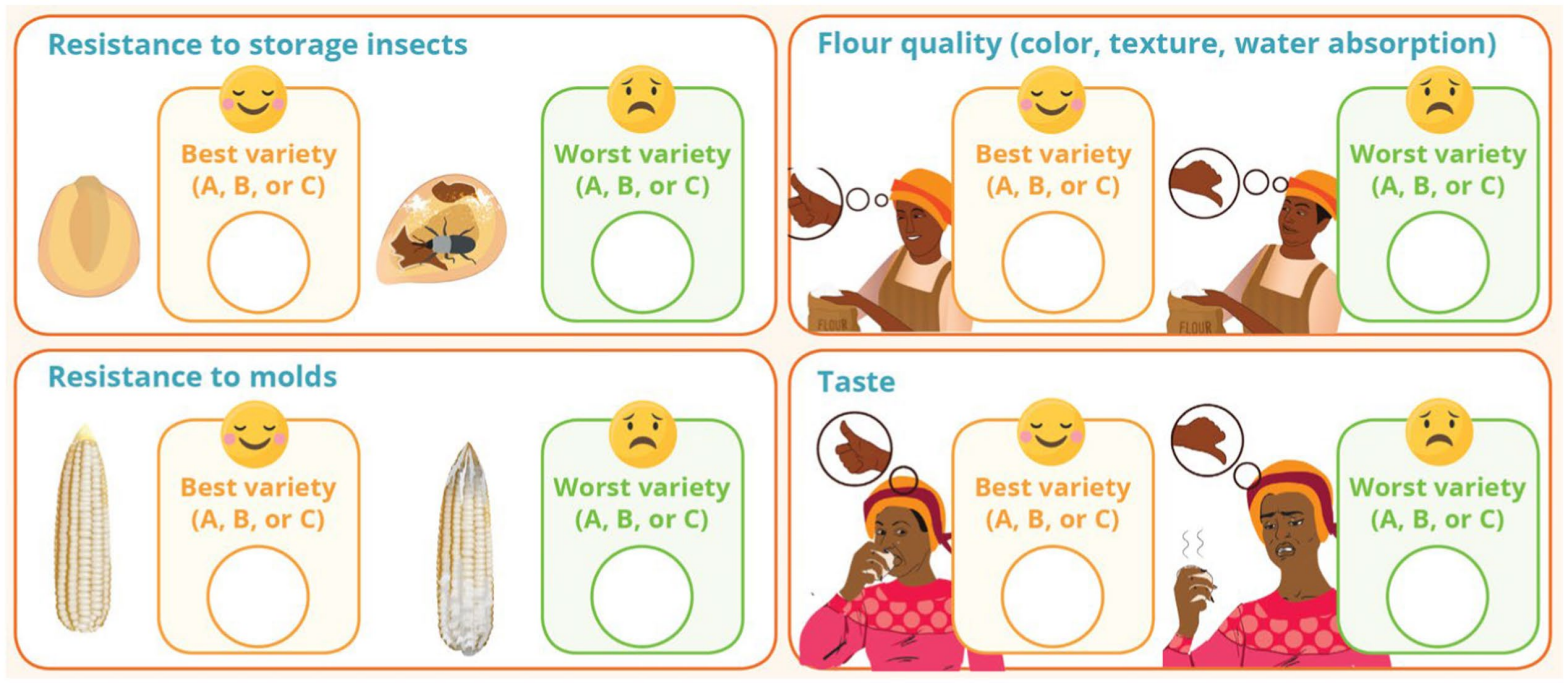
Participants in on-farm tricot trials were asked to rank their three varieties on agronomic and end-use traits.

The SPTA team also used the tricot trials to pilot new gender data collection methods. First, recognizing the prevalence of “joint” plot management, the gender researcher pushed for both men and women within households to participate in variety evaluations if both helped manage the trials. The gender researcher and breeding support specialist also advocated for expansion of the farmer evaluations to include processing, cooking, storage, and consumption traits (such as flour yield, flour quality, and taste), given that end-use traits are often a driver of gender-differentiated farmer preferences (Weltzien et al., 2019). CIMMYT breeding programs had not previously evaluated end-use preferences in on-farm maize trials (Figure 3).

Challenges emerged in verifying the managerial roles of different individuals within households and ensuring that the three trial varieties were stored, milled, and cooked separately across over 100 households. The CIMMYT and NARES team implementing the trials had limited capacity to refine approaches and improve data quality while attempting to scale-out the trials. As such, the team chose to abandon end-use trait evaluations after the pilot and narrowed evaluations to one individual per household (understanding end-use traits’ importance remained a priority in separate research by the CIMMYT team). In partnership with the 1000FARMS project, SPTA tricot trials expanded to 356 farmers (65% women) in Kenya in 2022, and 1,380 farmers (56% women) in 2023. The team’s experiences provided insights that helped other CIMMYT breeding projects adapt the tricot approach in ESA.

### 3.4. Role of gender in project activities

The SPTA project is a case in which gender considerations were present from the earliest stages of the project due to the commitment and training of breeding team members. However, collection and analysis of gender data was not included until SPTA1, when a donor mandated inclusion of gender considerations and funding. In SPTA2, the gradual expansion of social scientists’ involvement and funding increased the project’s attention to gender (Table 1).

**Table 1:** Methods and approaches in the Seed Production Technology for Africa (SPTA) project case.

Breeding activities undertaken	Gender focus in activities
Participatory varietal selection	Specific gender focus
Social science surveys	Specific gender focus
Trait preference studies	Specific gender focus
Mother-baby trials +citizen science	Specific gender focus

The discussion above highlights SPTA’s focus on gender through PVS, social science surveys, trait preference studies, and ultimately, trials using a citizen science approach. Social science surveys intentionally sought to uncover variation in management practices and seed choice between male- and female-headed households and male and female plot managers. Both PVS and tricot trials prioritized equal representation of men and women in evaluations. Social scientists and breeders centered gender in trial planning and analysis, working together to refined approaches.

The Seed Production Technology for Africa project’s growing gender focus culminated in adoption of the tricot approach. Tricot trials allowed for wider integration of gender considerations and diverse farmer needs, constraints, and preferences in the breeding process, rather than limiting gender assessments to *ex post* studies of technology acceptance. Tricot evaluations allowed farmers to assess varieties in their household and farm context, accounting for gender-related concerns such as labor requirements, end-use traits, and performance on low-fertility land. Although these were clear advantages of the tricot approach, the team also encountered challenges in collecting additional gender data, primarily in overseeing post-harvest storage and use and engaging with multiple respondents within households.

### 3.5. Changes to breeding processes and practices

Fundamentally, the integration of gender into SPTA’s breeding processes generated greater confidence in the FNP trait’s appeal. The pilot tricot trials provided experientially-derived gender preference data that showed no major gender-based differences in preferences to necessitate gender-intentional breeding. Rather, these methods enabled gender-responsive breeding—confirming that new FNP varieties do not generate negative gendered impacts and hold appeal under women’s and men’s real-world production conditions. As expanded tricot trials generate more data, clearer insights around gender-based preferences may emerge. For now, since the FNP trait showed particular promise for farmers with low yield expectations, the trait will be made available in key new stress tolerant hybrids to ensure that diverse farmer preferences do not limit access to or use of the FNP trait.

**Figure 4:**
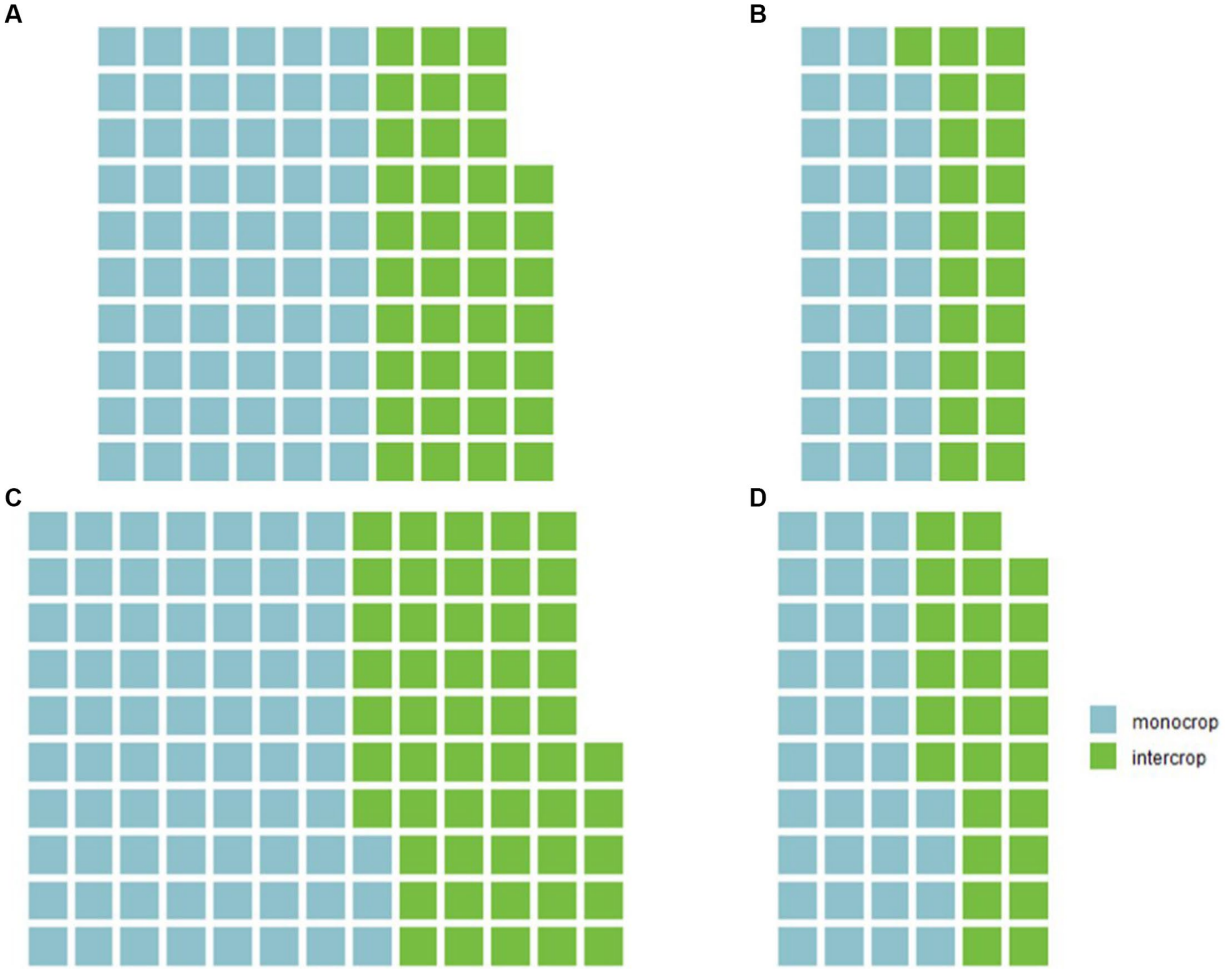
The number of **(A)** women farmers in Embu, **(B)** men in Embu, **(C)** women in Kiriyanga, and **(D)** men in Kiriyanga using monocropping and intercropping illustrate the diversity of farmer management in 2022 tricot trials in Kenya.

The Seed Production Technology for Africa project on-farm trial data did highlight wide variation in farmers’ management practices, including widespread use of intercropping among both women and men in Kenya (Figure 4). In combination with evidence of variation in farm practices along gender lines (Cairns et al., 2021a), these data validate the wider use of decentralized on-farm testing under farmer management.

Research in SPTA has influenced on-farm testing and advancement decisions within CIMMYT’s wider breeding programs, including expanded use of gender-disaggregated data. On-farm trials for product advancement throughout the maize breeding program have shifted to accommodate farmers’ preferred management practices. However, the long-term impacts of these changes to the breeding process are yet to be seen. While no varieties developed through SPTA have yet been released, FNP hybrids have entered the varietal release process in Kenya and South Africa.

## 4. Discussion

A number of “good practices” are evident in this case study, including trainings on gender for breeders; provision of funding for gender research; accounting for gender in identification of end users, breeding objectives, and variety design; and especially, attention to gender in farmer assessments and on-farm trial design. Each of these elements contributed to changing scripts and behaviors within institutions involved in maize breeding.

### 4.1. Creating space and budget for gender research and interdisciplinarity

The SPTA story drives home, perhaps above all else, the value of productive and respective collaborations across disciplines. In this case, the collaboration was enabled by PLs who embraced social scientists’ and gender researchers’ contributions and allocated funding to support them beginning in SPTA1. This allowed exploration of gender-relevant topics and testing of new gender-responsive variety evaluation tools.

Unfortunately, an openness to serious gender integration is not universal within breeding teams (Tarjem, 2023). Gender analyses are sometimes perceived to be overly complex, not adequately rigorous, and/or a distraction from the core goals of breeding. In the SPTA case, an understanding of the relevance of gender grew in part from prior trainings and exposure to robust gender data. This led actors to break with traditional institutional scripts about how breeders should interact with social scientists in breeding projects.

Opportunities for gender integration widened with a shift in control over on-farm trials to social scientists, an action that challenged existing behavioral scripts around breeding. Such shifts in dynamics can generate tension, but in SPTA, open collaboration between social scientists and the breeding team enabled the joint design of gender-responsive on-farm trials. The breeding team and social science teams each welcomed the knowledge and experience of the other, while the small size of the team enabled closer collaboration than is feasible in many larger scale projects. Furthermore, the PL and breeding program director added explicit mention of gender research to the job description for the female CIMMYT scientist on the project breeding team, empowering her to more actively and intentionally pursue these topics within her scope of work.

### 4.2. Accounting for gender in identification of end-users and market segments

The Improved Maize for African Soils and SPTA projects emerged from a specific desire to increase maize yields under low-input conditions. Even in the earliest project proposals, women and other resource-constrained farmers were identified as potential target demographics, and the SPTA PL’s dedication to serving the most vulnerable market segments strengthened this focus. After an early donor gender audit identified the need for added attention to gendered project impacts, project leaders increased the focus on farmer assessments and expanded research questions to include those relevant specifically to women (e.g., impacts of the FNP trait on seed recycling).

### 4.3. Accounting for gender in breeding objectives

From the earliest stages of the project, breeding varieties for performance under low-input conditions was a priority—initially through on-station trials and later through on-farm trials. In breeding, on-station trials are critical to ensuring high repeatability while allowing many varieties to be screened together. The focus of SPTA trials was to confirm performance and acceptance of the trait within the target population of environments (TPE), and later to engage farmers as citizen scientists. The breeding team actively took up recommendations of the donor gender audit, engaging gender specialists and other researchers to explore differences in management and resource constraints. This allowed for refinement of on-farm testing protocols, culminating in a design geared specifically toward understanding performance under diverse management practices and capturing farmer preferences in their personal household context.

### 4.4. Accounting for gender in variety design decisions

Gender integration in SPTA was not focused on breeding separate varieties for women and men, but rather on validating the gender-responsiveness and utility of the FNP trait for resource-poor farmers. Recognizing that men and women may seek a range of maize varieties for various reasons, the breeding team prioritized inclusion of the FNP trait into key female lines developed for stress prone environments. Men and women farmers were then allowed to evaluate FNP hybrids on their own farms to ensure their other variety needs and priorities were met. However, without clear indications of differing preferences, there are no signs that varieties specifically targeted at women are necessary.

### 4.5. Accounting for gender in on-farm trial design

A key innovation in the SPTA project was partnership between the breeding and social science teams in developing gender-responsive on-farm testing methods. These approaches ensured, initially, that centralized on-farm trials were representative of management by women and resource-poor farmers. Later, on-farm trials were redesigned to be decentralized, inclusive, participatory, and reflective of diverse farmer management. In that sense, SPTA stepped beyond the gendered trait and variety assessments typically used to assess variety acceptance in breeding programs (e.g., passive PVS). Increased participation of men and women farmers in the SPTA breeding process turned farmers from relatively passive recipients of new technologies into active developers of those technologies; citizen science approaches allowed for farmer evaluation of varieties in their real-world context. These changes also increased the breeding team’s confidence that FNP varieties respond to women’s needs and priorities.

### 4.6. Accounting for gender in farmer evaluations

Recognizing the potential benefits of the SPTA technology to women, SPTA prioritized equitable participation of men and women as on-farm trial hosts and in PVS, yielding over 50% participation by women and included many resource-poor farmers. Although the social science team piloted inclusion of gender-relevant end-use traits in tricot evaluations, these assessments proved too challenging to manage at scale with available capacity; other projects have found success in more structured consumer testing via home preparation using a tricot approach (Moyo et al., 2021). Still, given the participation by a diversity of farmers, the project breeding team gained increased confidence about the performance and appeal of FNP varieties under realistic farmer management conditions, including women’s unique management practices.

### 4.7. Lessons learned and case study limitations

Central to gender integration in this project was greater involvement of social scientists, including gender researchers, in on-farm trial design and management. The handover of on-farm trials to the social science team was unprecedented within maize breeding programs. There are many reasons why breeders might be reluctant to relinquish control over trials, including a desire to standardize approaches across projects, concern that social science teams and farmers lack the experience or technical knowledge to implement effective on-farm trials, or reticence to reform a system that has functioned adequately for decades in developing improved germplasm. Territoriality between breeders and social scientists may be worsened by programmatic divisions within research institutes; in this case, gender research fell under the purview of a program that is separate from the breeding program.

Another challenge in shifting control over on-farm trials involved trade-offs between researcher-managed trials and citizen science approaches. On-farm trials in breeding programs must provide evidence that new varieties perform in the TPE. Researcher-managed trials may provide more internally valid varietal comparison data to guide breeders’ advancement decisions, although this has been questioned (Kool et al., 2020). Citizen science approaches generate more realistic performance data and have higher external validity—a growing priority for donors, breeders, and social scientists working to understand adoption. Generating robust data requires either highly standardized on-farm protocols or extensive and resource-intensive on-farm trial networks (e.g., tricot trials). Although the decentralized on-farm trial approach in SPTA has proved useful for validating FNP varieties, including gender-responsiveness, these data have not yet been used in maize variety release decisions in ESA, which still rely on national performance trials.

The tricot approach has also introduced new challenges for NARES field staff who have typically implemented researcher-managed on-farm trials. Tricot trials are often more complicated to oversee than standardized trials because they hand over management to smaller-scale, resource-poor farmers without experience hosting trials. Indeed, some data quality concerns persisted in SPTA’s on-farm trials, but the project team was actively developing and implementing methods to improve data quality (e.g., use of multiple tiers of supervision, spot-checking, and distribution of visually detailed instruction booklets, variety scorecards, and storage bags). Transitioning on-farm trials to more participatory approaches requires capacity building and collaborative design processes that balance these trade-offs and acknowledge required changes to institutional scripts.

The heightened involvement of social scientists in SPTA required breeding team members to make space (including financially) for collaborators to conduct research and implement changes. In this case, this was enabled by specific actors, including the SPTA’s PL—someone deeply committed to serving the most vulnerable farmers and trained to think about farmer heterogeneity. He felt that social scientists rather than breeders were those best trained to assess seed demand and sought to strengthen their role in the project, in a departure from existing institutional scripts around breeding. Other project team members, two of whom were women, and one of whom was independently interested in gender integration, also embraced greater engagement with social scientists. It is worth noting that the PL’s interest in gender emerged from voluntary participation in trainings and a colleague’s presentation of robust, quantitative gender data, which captured his attention in ways that other gender discussions never had. This is an excellent example of a social scientist researcher using compelling quantitative data to speak persuasively to collaborators in the biophysical sciences, illustrating the importance of the type and quality of gender data that gender researchers produce and share with breeder colleagues.

Breeding projects seeking to achieve similar gender integration can take several lessons from this case. This includes the value of seeking out allies in breeding and social science teams who are willing to collaborate, test innovative approaches, and critically examine entrenched behavioral scripts in breeding institutions. Identifying such allies is not always easy, and building institutional capacity for and commitment to interdisciplinarity is critical. This includes, for example, ensuring that breeders are regularly exposed to accessible, carefully thought-through trainings related to gender and farmer behavior, and that social scientists in crop research institutes have adequate understanding of breeders’ data needs and decision-making processes. In the world of crop improvement, such collaboration and interdisciplinarity is not always rewarded. Social scientists are often pressured to generate meaningful independent research in their fields, so collaborations with breeders may not advance their careers. Breeders’ success, meanwhile, is measured in reference to trials with adequate heritability, which means trials’ internal validity is paramount while reaching under-served populations may go unrewarded. These incentive structures must be examined to ensure that breeders’ and social scientists’ shared goal of effective product development is prioritized. As seen in this case, breeders’ active partnership with social scientists in on-farm trials may increase confidence in collaboration and trust across project teams.

Finally, the SPTA case highlights the value in starting small. This was a limited project within a larger breeding portfolio where a close team could build productive partnerships. By first collaborating to pilot and develop proof of concept for new models, the team began reforming long-standing practices. Ultimately, the project team’s willingness to collaborate generated important proof of concept, know-how, and protocols that have helped shift how on-farm trials in other maize breeding projects, all of which are managed by breeding teams, are designed. Shifting the institutional scripts governing gender and breeding is a sea change and will not happen overnight, but the actions and interactions of a dedicated group of multidisciplinary collaborators may start the process.

## Data availability statement

The original contributions presented in the study are included in the article/supplementary material, further inquiries can be directed to the corresponding author.

## Author contributions

RV: Conceptualization, Funding acquisition, Investigation, Writing – original draft, Writing – review & editing. JC: Conceptualization, Funding acquisition, Investigation, Project Conceptualization, Funding acquisition, Project administration, Supervision, Writing – review & editing. FM: Investigation, Writing – original draft, Writing – review & editing. GM: Investigation, Writing – review & editing, Data curation. EH: Investigation, Writing – review & editing, Data curation. DL: Investigation, Writing – review & editing. KM: Investigation, Writing – review & editing. SC: Investigation, Writing – review & editing, Funding acquisition. SW: Investigation, Writing – review & editing. VW: Investigation, Writing – review & editing.
